# Etymologia: Oropouche Virus

**DOI:** 10.3201/eid2405.ET2405

**Published:** 2018-05

**Authors:** Ronnie Henry, Frederck A. Murphy

**Keywords:** etymologia, Oropouche virus, viruses, mosquitoes, midge, Culicoides paraensis, three-toed sloth, Vega de Oropouche, Trinidad, Trinidad and Tobago

## Oropouche [oʹro-pooʺche] Virus

In September 1955, a virus was isolated from a 24-year-old forest worker from the community of Vega de Oropouche, near the town of Sangre Grande, on the island of Trinidad (country: Trinidad and Tobago), who presented with fever, backache, and cough, which resolved spontaneously after 3 days. The virus was isolated from the patient’s blood by intracranial inoculation of suckling mice at the Trinidad Regional Virus Laboratory. Five years later, the virus was isolated from *Coquillettidia venezuelensis* (Theobald) mosquitoes collected ≈ 30 miles away in the Bush Bush Forest. The urban vector was later identified as the midge *Culicoides paraensis*, but the sylvatic vector remains unknown. Virus has been isolated from the three-toed sloth, which is believed to be involved in the sylvatic transmission cycle.

The virus was shown to be unique but antigenically related to Simbu virus, which had recently been described from South Africa. It therefore became a member of the large family of bunyaviruses.1

The virus, Oropouche virus, named in keeping with the tradition of designating arboviruses by using local geographic names, stems from the name of the village, a nearby swamp (wetland), and river. It derives from an Amerindian word, but the ancient meaning of the word is not clear.

Oropouche virus has since proven to be one of the most common arthropodborne viruses infecting humans in the tropics of the Western Hemisphere. Clinical signs of infection include headache, myalgia, arthralgia, and chills; no deaths have been reported. It has been estimated to have infected more than half a million persons in Brazil alone, and there have also been large outbreaks in Panama and Peru. In keeping with the recent emergence of other arboviruses such as West Nile, chikungunya, and Zika viruses, Oropouche virus is a candidate for possible further urban spread and therefore warrants increased surveillance and diagnostics.

## References

[1] Anderson CR, Spence L, Downs WG, Aitken TH. Oropouche virus: a new human disease agent from Trinidad, West Indies. Am J Trop Med Hyg. 1961;10:574–8. 10.4269/ajtmh.1961.10.574 13683183

[2] Travassos da Rosa JF, de Souza WM, Pinheiro FP, Figueiredo ML, Cardoso JF, Acrani GO, et al. Oropouche virus: clinical, epidemiological, and molecular aspects of a neglected orthobunyavirus. Am J Trop Med Hyg. 2017;96:1019–30. 28167595 10.4269/ajtmh.16-0672 PMC5417190

[3] Virus Taxonomy. V. The Classification and Nomenclature of Viruses. The Online (10th) Report of the International Committee on Taxonomy of Viruses (ICTV) [cited 2018 Mar 12]. https://talk.ictvonline.org/taxonomy/

